# Fractal Analysis of Permeability of Unsaturated Fractured Rocks

**DOI:** 10.1155/2013/490320

**Published:** 2013-03-27

**Authors:** Guoping Jiang, Wei Shi, Lili Huang

**Affiliations:** ^1^Earthquake Engineering Research Test Center, Guangzhou University, Guangzhou 510405, China; ^2^Ningbo Polytechnic, Ningbo, Zhejiang 315800, China

## Abstract

A physical conceptual model for water retention in fractured rocks is derived while taking into account the effect of pore size distribution and tortuosity of capillaries. The formula of calculating relative hydraulic conductivity of fractured rock is given based on fractal theory. It is an issue to choose an appropriate capillary pressure-saturation curve in the research of unsaturated fractured mass. The geometric pattern of the fracture bulk is described based on the fractal distribution of tortuosity. The resulting water content expression is then used to estimate the unsaturated hydraulic conductivity of the fractured medium based on the well-known model of Burdine. It is found that for large enough ranges of fracture apertures the new constitutive model converges to the empirical Brooks-Corey model.

## 1. Introduction

Modeling water flow in unsaturated fractured rocks has received considerable attention in the last two decades. One of the main reasons for focussing on the study of liquid flow in this type of media is that spontaneous capillary imbibition is an important fundamental phenomenon existing extensively in a variety of processes such as oil recovery, polymer composite manufacturing, soil science, and hydrology. The other reason is that deep disposal in crystalline rocks is considered to be an effective mean of isolating radioactive wastes from the biosphere. The study of basic transport processes has long been recognized because of which one can heighten the comprehensive understanding of physical phenomena, such as permeability [1, 2], heat transfer [3–5], and sorption [6, 7]. Many parameters such as the porosity, size of pore, and tortuosity of capillaries are very important for fluid flow in hard rocks. These parameters, however, are closely related to the geometric architecture of hard rocks. Cai et al. have analyzed the natural fractured trace maps representing a wide variety of scales, geological settings, and lithologies [8]. Cai and Yu have reported the density of different sized fault segments within the San Andreas fault zone which is fractal [9]. The distributions of fracture aperture and fracture spacing are self-similar over a well-defined range of apertures in the Cajon Pass scientific drill hole which was found by Barton and Zoback [10]. Based on the assumption that the fracture pattern is self-similar, the Sierpinski carpet was often employed to simulate porous media [11]. The conceptual constitutive model proposed by Wu and Yu [12] had used its fractal dimension to the parameters of the Brooks-Corey constitutive model [13] through the Sierpinski carpet. A Sierpinski space was also adopted to characterize the spatial distribution of a drainage network in the Gardon basin, France [14].

 From the above review it is shown that a mechanistic model has not yet been established. In this paper, we attempt to develop a mechanistic model for unsaturated flow in fractured hard rocks based on the method using the specific fractal to describe fractured rock. The expressions of the proposed constitutive model are closed form and easy to evaluate. Another important feature, the tortuosity fractal dimension *D*_*T*_, which affected other model parameters and should not be neglected while it was often neglected in the past investigation, is considered.

Now fractal theory has a wide variety of applications in sciences and engineering fields such as thermal science [15–19], fluid science [1, 2, 6], and industrial construction engineering [20]. For example, Moussa [2] has systematically investigated the transport of porous media based on fractal theory. The work [2] is open. Xiao et al. [3–5, 15, 16, 18, 19] have done much outstanding work on heat transfer of fluids by using fractal technique. In our work, we derive the analytical expressions for the relative hydraulic conductivity of fractured rock while taking into account the effect of pore size distribution based on the fractal geometry theory. 

## 2. Construction of the Fractal Model

**Figure 1:**
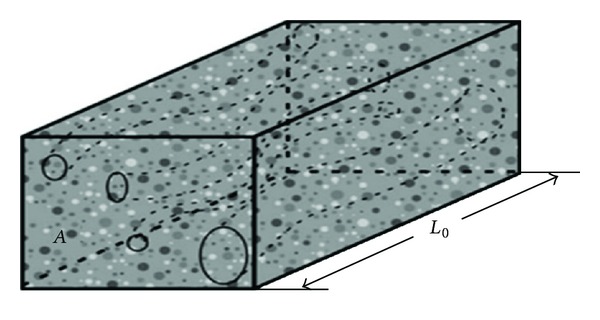
The model presented.

The model is presented in Figure 1. It has been shown that the cumulative size distribution of contact spots on engineering surfaces is similar to islands on earth and pores in porous rock which follows the fractal scaling law [2].

(1) $$\begin{array}{c} N\left(L\geq D_{n}\right)={\left(\frac{D_{n,\max}}{D_{n}}\right)}^{d_{f}}, \end{array}$$

where *d*_*f*_ is the fractal dimension for pores, *D* is diameter, *L* is the length scale, and *N* is the total number of pores whose sizes equal to and greater than *D*_*n*_. The number of pores whose sizes range from *D*_*n*_ to *D* + *dD*_*n*_ is

(2) $$\begin{array}{c} -dN=d_{f}D_{n,\max}^{d_{f}}D_{n}^{-(d_{f}+1)}dD_{n} \end{array}$$

when water flow through the pores of porous rock, the capillaries may be tortuous. These tortuous capillaries could be expressed by fractal equation [21]. Consider

(3) $$\begin{array}{c} L_{a}\left(D_{n}\right)=L_{0}^{D_{T}}D_{n}^{1-D_{T}}, \end{array}$$

where *D*_*T*_ is the tortuosity fractal dimension and lies in the range 1 < *D*_*T*_ < 2, which represents the extent of convoluted ness of capillary pathways for fluid flow through a medium. Note that for a straight capillary path *D*_*T*_ = 1, and a higher value of  *D*_*T*_  corresponds to a highly tortuous capillary. Let the diameter of a capillary in the medium be *D*_*n*_ and let its tortuous length along the flow direction be *L*_*a*_(*D*_*n*_). *L*_0_ is representative length of channels. With a straight capillary, *L*_*a*_(*D*_*n*_) = *L*_0_. The total volume of pores from *D*_*n*,min⁡_ to *D*_*n*,max⁡_ can be obtained from (1) as

(4) vtol=∫Dn,min⁡Dn,max⁡π4Dn2 La(−dN)=∫Dn,min⁡Dn,max⁡π4Dn2 LadfDn·max⁡df Dn−(df+1)dDn=π4(3−df−DT)L0DTdfDn·max⁡3−DT×(1−(Dn,min⁡Dn,max⁡)(3−df−DT)),

where *v*_tol_ is the whole volume of the pores, (−*dN*) is given by (2). 

Similarity to the state above, the volume *v*_(*D*_*n*,min⁡*x*_<*D*_*n*_)_of pores/particles from *D*_*n*_ to *D*_*n*,max⁡_ can be obtained from (1) as

(5) v(Dn,min⁡<Dn)  =π4L0DTdfDn·max⁡df∫Dn,min⁡xDn,Dn2−df−DTdDn  =π4(3−df−DT)L0DTdfDn·max⁡df(Dn3−df−DT)|Dn,min⁡Dn,.

The effective saturation *S* of volume *v*_(*D*_*n*,min⁡*x*_<*D*_*n*_)_ can be obtained. Consider

(6) Sv(Dn,min⁡<Dn)vtol|Dn,min⁡Dn,max⁡(Dn3−df−DT)|Dn,min⁡Dn,(Dn3−df−DT)Dn3−df−DT−Dn,min⁡3−df−DTDn,max⁡3−df−DT−Dn,min⁡3−df−DT.

Assuming the immiscible fluid flow in reservoir rocks, three relatively important forces are considered: the capillary pressure can be expressed. Consider

(7) $$\begin{array}{c} p=\frac{2\sigma \cos\left(\beta \right)}{\rho gD_{n}}, \end{array}$$

where *σ* is surface tension between the wetting and non-wetting fluids, *ρ* is the water density, *g* is the gravity acceleration, *D*_*n*_ is diameter of a pore, and *β* is contact angle between the extraneous water and solid.

The relative permeabilities are usually expressed in terms of water saturation *S*_*w*_. The saturation curve for the proposed model of fractured rock was derived. Consider

(8) $$\begin{array}{c} S_{w}=\frac{p_{c}^{3-d_{f}-D_{T}}-p_{c\min}^{3-d_{f}-D_{T}}}{p_{c,\min}^{3-d_{f}-D_{T}}-p_{c,\max}^{3-d_{f}-D_{T}}}, \end{array}$$

where

(9) $$\begin{array}{c} p_{c\max}=\frac{2\sigma \cos\left(\beta \right)}{\rho gD_{n,\min}},\;\;p_{c\min x}=\frac{2\sigma \cos\left(\beta \right)}{\rho gD_{n,\max}}. \end{array}$$

## 3. The Permeability of Unsaturated Fractured Rocks

The Burdine and Mualem models are the two most widely used models predicting relative hydraulic conductivity. For the particular state of flow in fractured hard rock, the Burdine model seems to be more consistent and to be often adopted. The expression of the Burdine model is

(10) $$\begin{array}{c} K_{D}\left(s\right)=s^{2}\frac{\int_{0}^{s}\left(ds/p_{c}^{2}\right)}{\int_{0}^{1}\left(ds/p_{c}^{2}\right)}, \end{array}$$

where *K*_*D*_(*s*) is the relative hydraulic conductivity. Inserting (8) to (10) we obtain the following form for *K*_*D*_(*s*):

(11) KD(s)=(pcdf+DT−3−pcmax⁡df+DT−3pc,min⁡xdf+DT−3−pcmax df+DT−3)2×pcdf+DT−5−pcmax⁡df+DT−5pcmin⁡xdf+DT−5−pcmax⁡df+DT−5.

The expressions of (6) and (10) represent the proposed constitutive model for fractured hard rocks. Note that all model parameters are determined by geometric parameters of *D*_*n*,max⁡,_, *D*_*n*,min⁡*x*_ and residual water content *β*.

The novel constitutive model has some similarities with the well-known Brooks-Corey model, which is *S*_0_ = (*p*_*c*_/*p*_*d*_)^−*λ*^ and *K*_*D*_(*s*) = *S* ^3+*λ*/2^, where *p*_*d*_ is the reciprocal of air entry pressure and *λ* is a model parameter related to pore size distribution.

**Figure 2:**
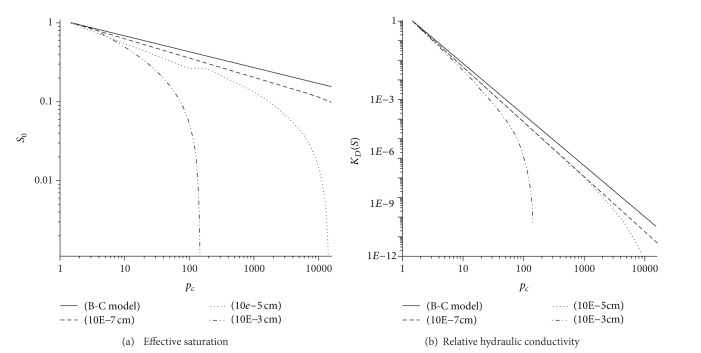
Comparison between the Brooks-Corey model and the new relations for different ranges of fracture apertures when *D*_*T*_ = 1.

When *D*_*T*_ = 1, *λ* = 2 − *d*_*f*_, and *p*_*c*,max⁡_ ≪ *p*_*c*,min⁡*x*_, *p*_*c*,max⁡_ is neglected here, the model derived here is simplified to the Brooks-Corey model. Comparisons of the proposed and the Brooks-Corey models for three different ranges of fracture apertures are depicted in Figure 2. The assumed parameters are *d*_*f*_ = 1.8, *D*_*n*,max⁡_ = 10^−1^ cm, and *D*_*n*,min⁡*x*_ = 10^−3^ cm, 10^−5^ cm, and 10^−7^ cm. According to Figure 2, the Brooks-Corey model seems to be adequate to describe the hydraulic properties of fractured rocks for large ranges of fracture apertures and low values of pressure head.

**Figure 3:**
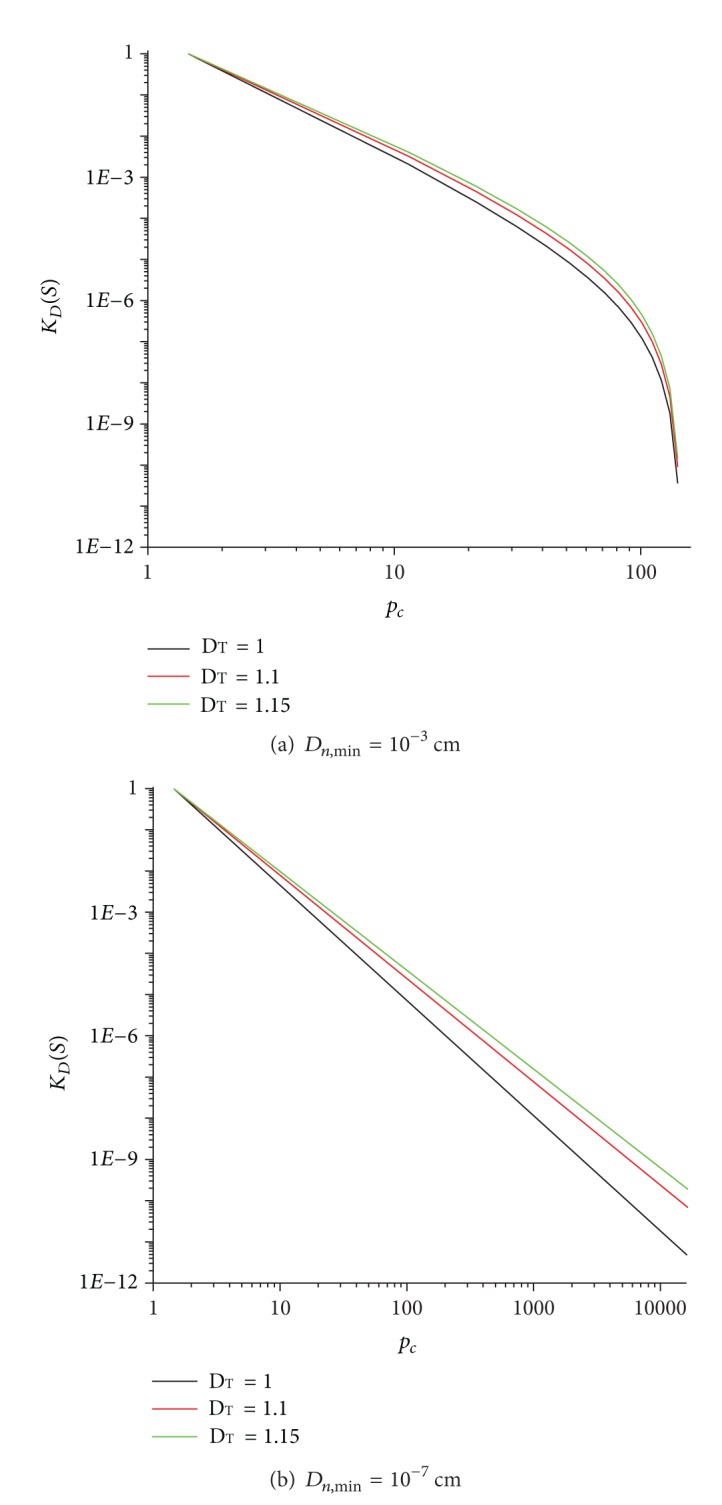
The new relations for *K*_*D*_(*S*) and *D*_*T*_.

**Figure 4:**
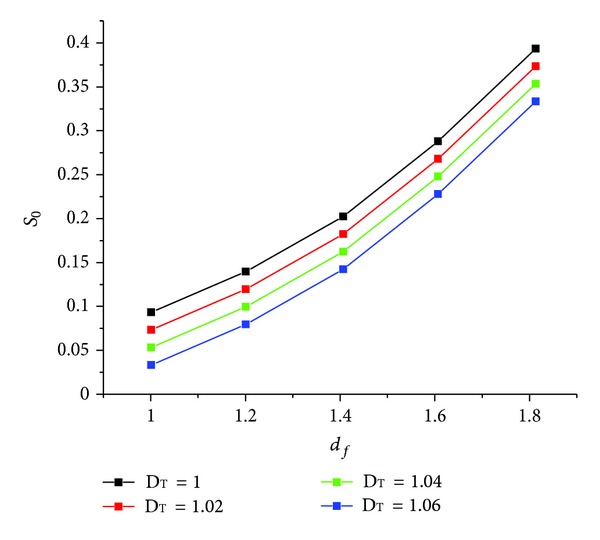
The new relations for *S*_0_ and *d*_*f*_.

 The relationship between the fractal dimension and effective saturation is very important in the study of unsaturated flow in fractured hard rocks. The geometric parameters and physical constants used for the analysis are *β* = 0, *σ* = 72.25 dy/pa. In order to analyze the influence of the fracture density we consider different values of *D*_*T*_. With (8) and (11), the relationship is obtained (Figure 3). With the physical constants *D*_*n*_ = 0.1 cm,  *D*_*n*·max⁡_ = 1 cm, and *D*_*n*·min⁡*x*_ = 0.01 cm, the relationship between *d*_*f*_ and *S*_0_ can also be determined (Figure 4).

 In order to test the proposed invasion depth model, it is crucial to correctly determine the fractal dimensions *D*_*T*_ and *d*_*f*_.

According to the following formula proved recently by Yu and Li [22, 23],

(12) $$\begin{array}{c} \phi ={\left(\frac{D_{n,\min}}{D_{n,\max}}\right)}^{D_{E}-d_{f}}, \end{array}$$

where *D*_*E*_ is the Euclidean dimension and *D*_*E*_ = 2 is used in this work. The maximum pore diameter can be calculated based on the model of square arrangement of particles.

According to the following formula proved recently by Jiangchao Cai, where *K* is permeability, *k* is Kozeny constant, which considers the tortuosity of capillaries and pore non-uniformity. And the parameters *K* and *k* are all proved [23]. Consider

(13) $$\begin{array}{c} D_{T}=1+\frac{\ln t_{\text{av}}}{\ln L_{0}/t_{\text{av}}},\\ D_{n,\max}=\frac{2\left(1-\phi \right)}{\phi }\sqrt{\frac{Kk}{\phi }}\left(\sqrt{\frac{\phi }{1-\phi }}+\sqrt{\frac{\pi }{4\left(1-\phi \right)}}-1\right), \end{array}$$

where

(14) $$\begin{array}{c} t_{\text{av}}=\frac{1}{2}\left[1+\frac{1}{2}\sqrt{1-\phi }+\frac{\sqrt{{\left(\sqrt{1-\phi }-1\right)}^{2}+\left(\left(1-\phi \right)/4\right)}}{1-\sqrt{1-\phi }}\right],\\ \frac{L_{0}}{t_{\text{av}}}=\frac{d_{f}-1}{2}\sqrt{\frac{1-\phi }{\phi }\frac{\pi }{d_{f}\left(2-d_{f}\right)}}\frac{D_{n,\min}}{D_{n,\max}}. \end{array}$$

**Figure 5:**
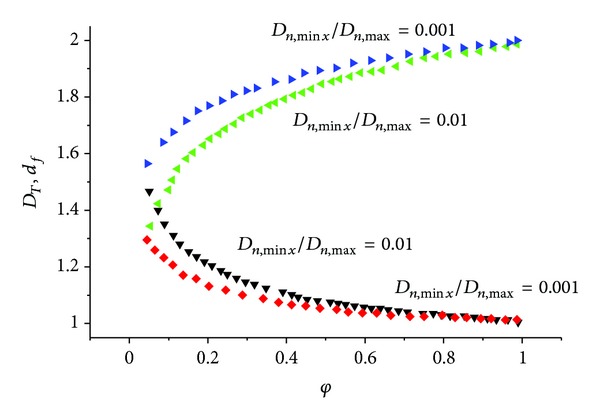
The relationship among *ϕ*,*D*_*T*_, and *d*_*f*_.

From (12) and (13), the relationship among *ϕ*, *D*_*T*_, and *d*_*f*_ is determined (Figure 5).

## 4. Conclusions

In this paper, with the consideration of pore size distribution and tortuosity of capillaries, a new fractal model for relative hydraulic conductivity of fractured rock is developed. The derived constitutive model is an effort to understand and characterize unsaturated flow in fractured rocks. The expressions of water content and relative hydraulic conductivity curves have analytical closed forms. The parameters can be completely determined by the geometry of the fractal model. Every parameter of the proposed formulas of calculating relative hydraulic conductivity of fractured rock has a clear physical meaning. The fractal model can reveal the mechanisms of hydraulic conductivity for fluids flow through unsaturated rocks. The tortuosity fractal dimension *D*_T_ which affected other model parameters and should not be neglected while it was often neglected in the past investigation is considered in this paper.
